# Recombinant cystatin ingestion by *Diaphorina citri* reduces insect survival: insights into the HLB host–bacteria interaction (*D. citri*–*C*Las) focused on DcCathL studies

**DOI:** 10.3389/finsc.2026.1700002

**Published:** 2026-02-11

**Authors:** Sâmara V. Rocha, Chiara Bernardini, Poulami Sarkar, Chun-Yi Lin, Karina Fan, João Paulo R. Marques, Juan C. Cifuentes-Arenas, Maria Cristina S. Pranchevicius, Nelson A. Wulff, Fabrício J. Jaciani, Marcelo B. Cioffi, Daniel L. Stanton, Amit Levy, Andrea Soares-Costa

**Affiliations:** 1 Department of Genetics and Evolution, Federal University of São Carlos, São Carlos, SP, Brazil; 2 Department of Agriculture, Food Animal and Environment Di4A, Università degli Studi di Udine, Udine, Italy; 3 Entomology Department, University of California, Riverside, Riverside, CA, United States; 4 Citrus Research and Education Center, University of Florida, Lake Alfred, FL, United States; 5 Department of Basic Sciences (ZAB), School of Animal Science and Food Engineering, University of São Paulo, Pirassununga, SP, Brazil; 6 Department of Research and Development, Fund for Citrus Protection – Fundecitrus, Araraquara, SP, Brazil

**Keywords:** *Huanglongbing*, HLB, *Diaphorina citri*, *Candidatus* Liberibacter asiaticus, *C*Las, cystatins, cathepsins

## Abstract

Huanglongbing (HLB) is considered the most serious Citrus disease for which there are currently no effective control methods. The putative agents of HLB, *Candidatus* Liberibacter asiaticus (*C*Las), and the vector *Diaphorina citri*, are widespread in citrus regions, causing losses in citrus cultivation worldwide. Studying the interactions between *D. citri* and *C*Las can elucidate disease epidemiology and determine specific targets for HLB control. This work aimed to improve our understanding of the host–bacteria (*D. citri*–*C*Las) relationship, focusing on cysteine peptidase (DcCathL) and its inhibition by citrus cystatin (CsinCPI-2). In this study, a combination of qPCR, FISH, and immunolocalization techniques was employed to detect DcCathL genes or proteins in *C*Las-free or -infected *D. citri* samples. To verify the effect of DcCathL inhibition on insect survival, an artificial diet assay containing recombinant CsinCPI-2 was performed with infected and healthy *D. citri* nymphs and adults. Accordingly, a relative expression of *DcCathL* 1.23 times higher and an approximately 3.3 times greater *DcCathL* transcripts in gut tissue were demonstrated in *C*Las-infected compared to *C*Las-free insects. Furthermore, the presence of DcCathL protein was detected in the gut, ovary, and salivary glands of *D. citri*, concentrated at the peripheral regions of the cells. The fluorescence signal associated with DcCathL indicates that the gut of infected *D. citri* contains 4.81 times more protein than the gut of a healthy insect. Similarly, the protein levels in the salivary glands and ovaries of infected insects were 1.38 and 1.1 times higher, respectively, compared to those of *C*Las-free insects. The efficacy of the artificial diet in delivering the recombinant protein to the insect was demonstrated by the detection of green fluorescent protein (GFP) in the intestinal tract of nymphs and adults. Finally, the CsinCPI-2 demonstrated a substantial increase in mortality among *C*Las-free nymphs (df = 1, p < 0.0001) and *C*Las-free or *C*Las-infected adults (df = 1, p = 0.0001). Thus, the development of inhibitors that can disrupt the interactions between bacteria and vectors by blocking DcCathL activity may represent a promising strategy to prevent the spread of HLB disease.

## 1 Introduction

The Asian citrus psyllid (ACP) *Diaphorina citri* Kuwayama (Hemiptera: Liviidae) is the vector of the bacterium *Candidatus* Liberibacter asiaticus (*C*Las) (1, 2), an unculturable, Gram-negative, phloem-limited and alpha-proteobacteria (3). *C*Las is the putative causal agent of *Huanglongbing* (HLB), a highly destructive citrus disease (1, 4), for which there is no cure. *C*Las and the vector *D. citri* are widespread in the main citrus-growing regions worldwide and constitute a serious threat to citrus production (1, 5–7). When *C*Las colonizes the phloem of plants, it disrupts the distribution of photoassimilate compounds, leading to typical symptoms such as blotchy-mottled and yellowish/chlorotic leaves, smaller fruits, smaller plants, and more acidic juice (1).

*D. citri*, a sap-sucking insect, acquires *C*Las by feeding on the phloem of infected citrus plants. The disease spreads quickly as adults move short distances and fly between trees, spreading the bacterium to uninfected citrus trees (8, 9). The disease has spread rapidly, causing serious economic impacts worldwide (10). For example, 44.35% of the plants in the citrus belt of São Paulo and west/southwest of Minas Gerais state show symptoms of HLB (11), and in Florida, HLB is observed in nearly 100% of the plants. This has led to a substantial decline in productivity and an increase in production costs (10).

Bacterial acquisition occurs when an insect salivates into phloem sieve elements while feeding (12). The life cycle of insects can be divided into egg, nymph (with five molts), and adult stages (13). Whereas the nymph stage is particularly efficient at acquiring the bacterium, the adult who acquired the bacteria in this phase is significantly more effective at spreading it (8). If the *C*Las bacterium is acquired at the adult stage, its transmission is less effective (8). After bacterial acquisition, *C*Las seems to form a biofilm in the midgut of *D. citri* and then translocate to the hemolymph before reaching the salivary glands (14). However, the exact mechanism by which the bacterium evades the insect immune system is still unknown (14).

Understanding the relationships among vectors, pathogens, and hosts is crucial for providing insights into the epidemiology of plant diseases (15). While most animal pathogens cause diseases in their insect vectors (16), the interaction between plant pathogens and their vectors can vary significantly. Some plant pathogens may even improve the fitness benefits of their vectors (17–19). Vector-borne bacteria have developed complex strategies to interact with their insect vectors and host plants (20). The successful development of several molecular tools and artificial diet assays has allowed the study of proteins involved in important interactions in the pathogen–host relationship, shedding light on new biological processes that regulate such intracellular pathogens (20). Peptidases, defined as proteolytic enzymes that catalyze the hydrolysis of peptide bonds (21), play a significant role in various biological processes.

Some cathepsin enzymes belong to the group of papain-like cysteine peptidases (PLCPs) and have a potential role in insect development (21, 22). Strategies focused on blocking the properties of these cathepsins have been recently studied (23). In *D. citri*, DcCathL is highly expressed at the egg stage and in intestinal tissue (24), showing an increased expression level after exposure to *C*Las (25), suggesting that DcCathL plays an important role in embryonic development, digestion, and immune defense (23–25).

The investigation of specific inhibitors capable of disrupting the interactions between bacteria and vectors is a promising strategy to slow the spread of *C*Las (26). Accordingly, in viruses, protein interactions are highly important in infection and transmission by insects (27), indicating that viruses can indeed control vector behavior by influencing their interactions with host plants (28). It has been demonstrated that *D. citri* flavi-like virus (DcFLV) modulates the cellular and physiological functions of *D. citri*, promoting the acquisition of *C*Las at the nymphal stage and transmission at the adult stage. Such modulations may be associated with genes such as cathepsins, which are upregulated in viruliferous adult psyllids and downregulated in viruliferous nymphs (29). Although the relationships among plants, bacteria, and vectors are still poorly understood (30), these interactions are also vital in diseases involving bacterial infections (31) and should be exploited. Considering that the interactions of cysteine peptidase inhibitors (CPIs) with PLCPs play crucial roles in defense processes and the regulation of endogenous peptidases (32, 33) and that CPIs in pest control studies have been widely explored (34–37), CPIs can be considered promising tools for the development of effective methods to combat HLB.

This work aimed to improve our understanding of the host–bacteria (*D. citri*–*C*Las) relationship, focusing on cysteine peptidase (DcCathL) and its inhibition by citrus cystatin (CsinCPI-2). A significant difference was found in the DcCathL expression pattern in *D. citri* in the presence and absence of *C*Las bacteria, as well as differences in the survival rates of the nymph and adult groups fed an artificial diet containing cystatin compared with those of the control group. The possibility of exploiting specific CPIs to interfere with bacterial–vector–host interactions can support the development of a promising strategy for HLB disease management (38, 39).

## 2 Materials and methods

### 2.1 Insects and plant materials

Nymphs from *C*Las-free (*C*Las^–^) and *C*Las-infected (*C*Las^+^) colonies of *D. citri* were maintained under controlled conditions on *Murraya koenigii* and *Citrus macrophylla* plants, respectively, at the University of Florida (Citrus Research and Education Center – CREC; Lake Alfred – FL, USA). Adults from *C*Las^–^ and *C*Las^+^ colonies of *D. citri* were maintained under controlled conditions on *Murraya paniculata* and *Citrus macrophylla* plants, respectively, at Fundecitrus (Fund for Citrus Protection; Araraquara – SP, Brazil), reared as previously described (40).

### 2.2 RNA isolation and cDNA synthesis

RNA was extracted from a pool of five *D. citri* adults using Trizol (Invitrogen, CA, USA) according to the manufacturer’s instructions. The integrity of the RNA was analyzed on the basis of the rRNA pattern in a 1% agarose gel. The RNA purity was checked by absorbance ratios at A260/A280 and A260/A230 obtained via UV/Vis spectrophotometry with Biodrop Duo (Biochrom., United Kingdom). cDNA synthesis was performed via a First Strand cDNA Kit (Applied Biosystems, CA, USA) according to the manufacturer’s instructions.

### 2.3 *DcCathL* gene expression evaluation through quantitative PCR

Quantitative PCR (qPCR) was performed on a 7500 Fast Real-Time PCR system (Applied Biosystems, Waltham, USA) using Power Up™ SYBR Green Master Mix (2x) (Applied Biosystems, Waltham, USA) with the primer pairs DcCathL-F/DcCathL-R for *DcCathL* and DcGAPDH-F/DcGAPDH-R for *GAPDH* as a reference gene (**Supplementary Table S1**) (41). The SYBR Green Master Mix reaction was performed in a 15 μL reaction mixture containing 6 μL of SYBR Green (Applied Biosystems, Waltham, USA), 1 μL of each primer (10 μM), 1 μL of the appropriate cDNA template and 6 μL of water. The qPCR was carried out as follows for *DcCathL*: 50 °C for 2 min and 95 °C for 2 min; 40 cycles at 95 °C for 20 s; and 60 °C for 30 s. After the final PCR cycle, a melting curve analysis was performed to determine the specificity of the reaction. Six biological replicates were performed, each of which was tested in duplicate. The gene expression of *DcCathL* was evaluated via normalization, with *D. citri GAPDH* used as a reference gene (41). Relative expression levels were calculated via the 2^−ΔΔCt^ method (42).

### 2.4 Fluorescence *in situ* hybridization for *DcCathL*

*D. citri* adults were collected from *C*Las^+^ and *C*Las^–^ colonies in the greenhouse and incubated for 20 min on ice, after which the midguts were dissected (**Supplementary Figure S1**) in phosphate-buffered saline (1x PBS, pH 7.4). The PBS was removed after dissection, and 300 µL of Carnoy’s fixative was immediately added (chloroform: ethanol: glacial acetic acid, 6:3:1) to fix the midguts for 5 minutes. After fixation, the samples were then hybridized overnight in the dark in 1 mL of hybridization buffer (20 mM Tris-HCl pH 8.0, 0.9 M NaCl, 0.01% sodium dodecyl sulfate (SDS), 30% formamide) containing 10 pmol of *DcCathL* fluorescent probe (Cy5-DcCathL-5’: TCAGGCTCGTAGTACACACC-3’) in an adapted small humid chamber. Following hybridization, the midguts were transferred with an appropriate needle to a fresh microscope slide. The samples were stained with a solution of DAPI (Southern Biotech, Birmingham, USA) and visualized under a Leica SP8 multiphoton confocal microscope (Leica Biosystems, Wetzlar, Germany) equipped with a 40x oil immersion objective. The localization of the DAPI and Cy5-DcCathL fluorescent probes was visualized with excitation at 405 nm and 633 nm. The emission was detected at 457 and 667 nm. A no-probe sample was used as a negative control (43).

The absolute fluorescence intensity of the *DcCathL* gene in the gut (*C*Las^–^ and *C*Las^+^) was determined via ImageJ (FIJI) (44, 45), based on the Corrected Total Area Fluorescence (*CTAF*) = *Integrated Intensity – (ROI Area x Average Background Intensity)*. To quantify the change in *DcCathL* relative expression, the CTAF of the infected condition was normalized relative to the CTAF obtained for *C*Las^–^, generating the Relative Fluorescence (RF), expressed as follows: $RF\;=\;CTAF_{CLas^{+}}/CTAF_{CLas^{-}}$; *C*Las^–^ was normalized to 1.0.

### 2.5 DcCathL immunolocalization

Polyclonal DcCathL enzyme-specific antibodies were produced by immunizing rabbits at the Immunology Laboratory of the Biotechnology Centre of the Federal University of Rio Grande do Sul (UFRGS). This antibody was used to target the DcCathL protein in *D. citri* organs, and the *D. citri* insects were immobilized and dissected in the same way as described above. Thus, the midgut, ovary, or salivary gland dissected (**Supplementary Figure S1**) was fixed for 40 min in 4% paraformaldehyde, washed three times with 1x PBS, permeabilized with 0.1% Triton X-100 for 20 min at room temperature, washed in PBST (PBS + 0.5% Tween 20) and blocked overnight with PBST containing 1% BSA at 4 °C. The organs were incubated in blocking buffer (PBST + 1% BSA) containing the DcCathL antibody at a concentration of 1:50 for 2 h at room temperature. This was followed by three washes of 5 min in PBST and subsequent incubation in the dark with blocking buffer containing Alexa Fluor 568 (1:250) (Invitrogen, Waltham, Massachusetts, USA) for gut, ovary, and salivary gland tissues for 2 h at room temperature. The samples were rinsed again three times in PBST, and the organs were transferred to microscope slides, counterstained with DAPI (Southern Biotech, Birmingham, USA) and visualized under a Leica SP8 multiphoton confocal microscope (Leica Biosystems, Wetzlar, Germany) equipped with a 40x oil immersion objective or Leica SP5 confocal microscope (Leica Biosystems, Wetzlar, Germany) (46) for salivary gland analysis. The secondary antibodies used for DAPI, goat anti-rabbit Alexa Fluor™ 568 (Thermo Fisher Scientific, Oregon, USA), were visualized with excitations of 405 and 578 nm, respectively. The emission was detected at 457 nm and 603 nm. A sample containing only the Alexa Fluor™-conjugated secondary antibody was used as a negative control.

CTAF was calculated to determinate absolute fluorescence intensity of the DcCathL protein in the gut, ovary and salivary glands (*C*Las^–^ and *C*Las^+^) for each microscope images obtained in 10x magnification, through ImageJ (FIJI) ROI fluorescence measures (44, 45) and RF was obtained to quantify the change in DcCathL relative expression, as described previously.

### 2.6 Production of the recombinant protein CsinCPI-2 and GFP

The *CsinCPI-2* and GFP genes were previously cloned and inserted into the expression vector pET28a (Novagen, Darmstadt, Germany) (47, 48), and the recombinant plasmids were subsequently transformed into NiCo21 (DE3) competent *E. coli* cells (New England BioLabs, Ipswich, MA). Recombinant expression and purification of both CsinCPI-2 and GFP proteins were performed as previously described (47–49). Briefly, bacterial cells were grown at 37 °C and 200 rpm to an OD_600_ of 0.4–0.6. Then, IPTG (0.4 mM; Sigma–Aldrich, San Luis, Missouri, USA) was added to the culture, after which expression were induced for 4 h and 2 h, respectively. The recombinant proteins containing a His-tag were purified via Ni-NTA Superflow Resin (Qiagen, Valencia, CA, USA) following the manufacturer’s instructions. The heterologous expression results were verified by 15% SDS–PAGE as described in Laemmli (50). The purified fractions were dialyzed in 0.1x PBS (pH 7.4), and total proteins quantification were performed via the Pierce™ BCA Protein Assay Kit (Thermo Scientific, Rockford, IL, USA). Following quantification, the recombinant proteins were added individually to the sucrose solution (v/v) used for the artificial diet test.

### 2.7 Wipe-feeding membrane bioassay for *D. citri* nymphs

To evaluate the effects of the CsinCPI-2 protein on *C*Las^–^ and *C*Las^+^*D. citri* nymphs, we performed an artificial-feeding bioassay via the folded wipes method of Tavares and Bonning (51) with some modifications (**Supplementary Figure S2A**). Using this model of artificial diet for nymphs, Kimtech Science^®^ Kimwipes (Kimberly Clark, Irving, TX, USA) were folded eight times, cut into a rounded shape to fit in a mini petri dish (35 mm × 12 mm) and sterilized. A sterile 15% (w/v) sucrose solution containing 0.1% (v/v) green and 0.4% (v/v) yellow food dyes was prepared with CsinCPI-2 (440 µg/mL) to test the effects of the inhibitor on the psyllid, and a 15% (w/v) sucrose diet containing 0.1x PBS buffer was used as a control in the experiment. Independently, 700 µL of each treatment diet solution was applied on the surface of the folded wipes at a final rate of approximately 70 µL/cm^2^. The bioassay evaluation was performed with three technical replicates containing 11 insects each. The experiment was repeated for a total of four biological replicates. The evaluation lasted 72 hours, and mortality was recorded daily in each group. Additionally, the same sucrose mixture with food dyes was prepared with recombinant green fluorescent protein (822.91 µg/mL) (rGFP), and after the feeding period, the midgut from those insects was dissected as previously described and evaluated under a Leica SP8 multiphoton confocal microscope (Leica Biosystems, Wetzlar, Germany).

### 2.8 Sachet-feeding bioassay for *D. citri* adults

The effects of the CsinCPI-2 protein on *C*Las^–^ and *C*Las^+^*D. citri* adults were determined via the parafilm M^®^-sachet feeding method, as described by Galdeano et al. (52), with slight modifications. A sterile 30% (w/v) sucrose solution containing 0.1% (v/v) green and 0.4% (v/v) yellow food dyes was prepared with CsinCPI-2 (440 µg/mL) to test the effects of the cystatin on the psyllid. An equally concentrated 30% (w/v) sucrose solution containing 0.1x PBS buffer, instead of protein, was prepared as a control. Plastic tubes 2.5 cm in diameter × 2.5 cm in height were used to set up the feeding chambers. Ten adults were carefully transferred to each tube, and the top was covered with a Parafilm M^®^ membrane. A total of 350 µL of the artificial diet solution (CsinCPI-2 at 440 µg/mL or 0.1x PBS) was applied on top, and a second parafilm membrane was stretched to hold and allow even distribution of the solution within the sachet (**Supplementary Figure S2B**). To keep the humidity suitable for the insects in the “feeding chamber”, a similar Parafilm sachet containing sterile water was placed in the bottom part of the tube, in the same way as described above. The tubes were maintained under light at 25 ± 2 °C and 50 ± 5% relative humidity with a 14 h:10 h light: dark cycle. The assay was performed in five technical replicates and the insect mortality was recorded daily for 12 days (264 h).

### 2.9 Statistical analysis

Data on *DcCathL* gene expression in *C*Las^+^ and *C*Las^–^ psyllids were subjected to a t test. The mortality rates of the *C*Las^+^ and *C*Las^–^ psyllids in the CsinCPI-2 and control groups were analyzed via Cox proportional hazards regression. The normality of the data was previously checked via the Shapiro–Wilk test. A significance level of *p* < 0.05 was used for all analyses, which were carried out in RStudio software v. 2023.6.2.561 (53).

## 3 Results

### 3.1 Gene expression analysis by qPCR and FISH for *C*Las^+^ and *C*Las^–^*D. citri*

**Figure 1:**
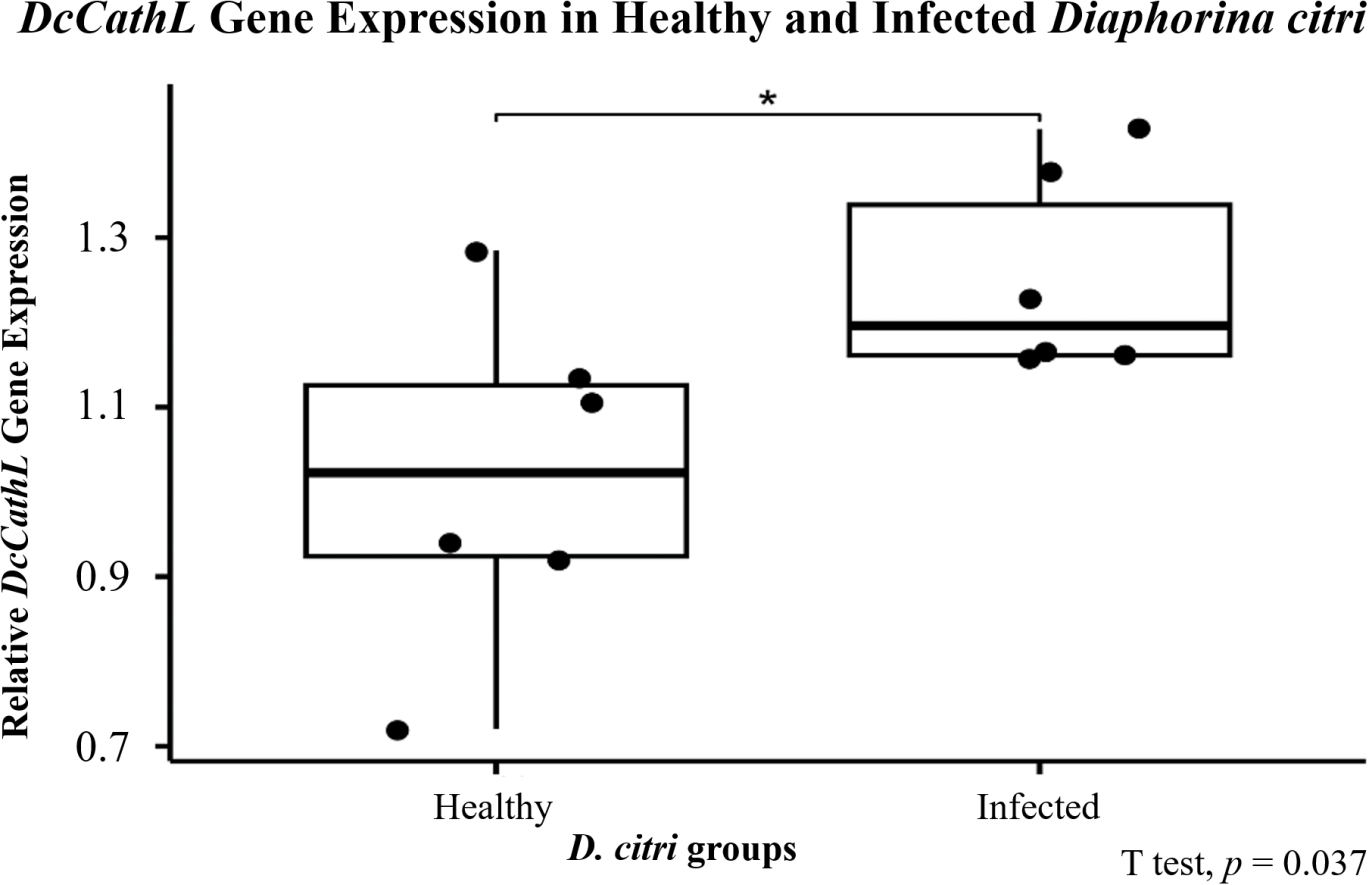
Relative *DcCathL* gene expression of healthy (*C*Las^−^) and infected (*C*Las^+^) *D. citri*. The boxplot represents the gene expression of *DcCathL* normalized to *D. citri GAPDH* and evaluated via qPCR. Statistical analysis was performed via RStudio (v. 2023.6.2.561) software. Significant differences among the means are indicated by * (*p* < 0.05).

RT–qPCR was performed to confirm the relative expression level of the *DcCathL* gene in the psyllids *C*Las^+^ and *C*Las^–^. qPCR analyses revealed that the expression of *DcCathL* was 1.23-fold-higher in *C*Las^+^ psyllids compared with that in *C*Las^–^ (df = 8.24, *p* = 0.037 (**Figure 1**)).

**Figure 2:**
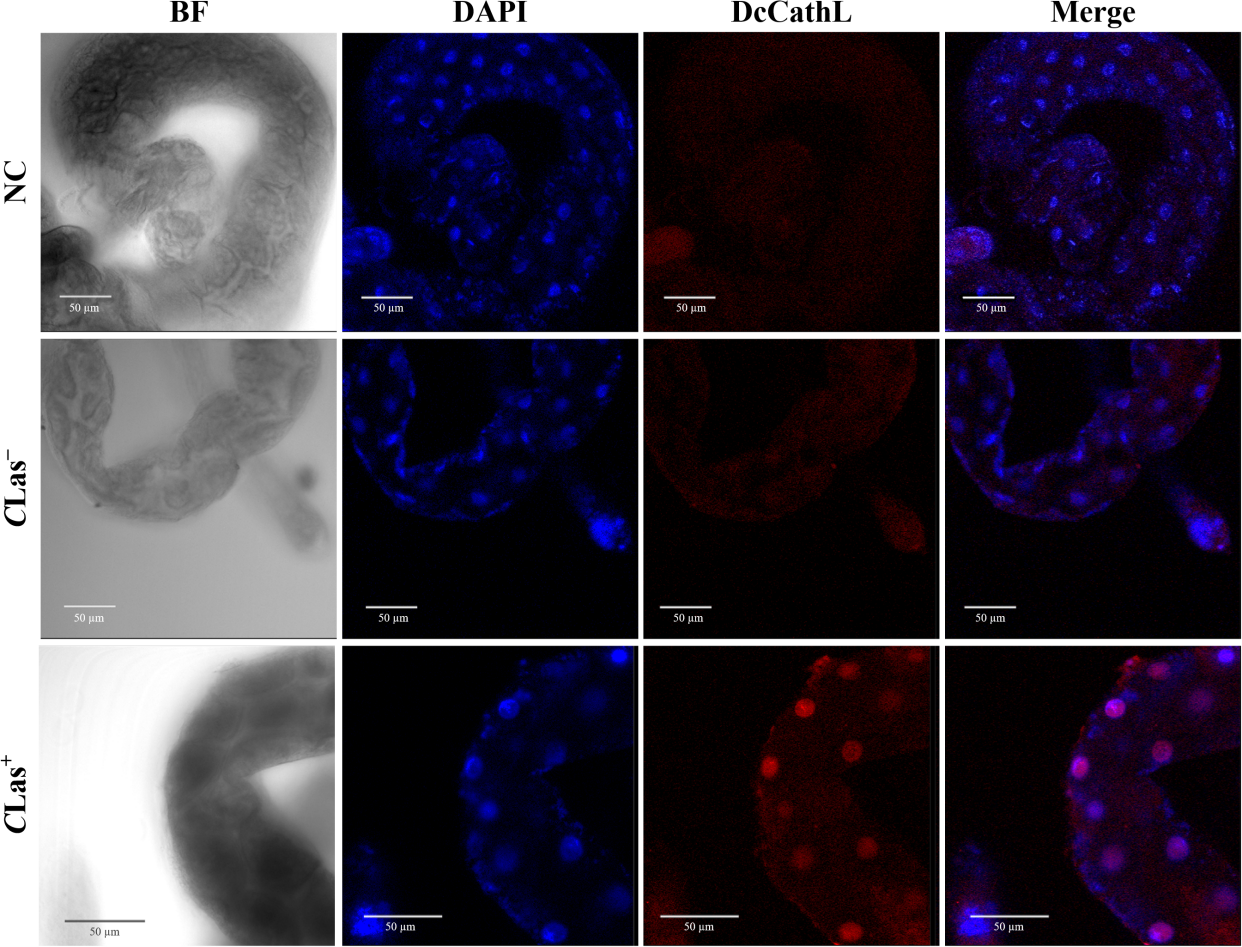
Fluorescence *in situ* hybridization (FISH) to detect *DcCathL* transcripts in gut tissues dissected from healthy (*C*Las^−^) and infected (*C*Las^+^) *D. citri*. BF, Bright field showing the morphology of the midgut tissue isolated from *D. citri;* DAPI, blue staining of the gut cell nuclei; *DcCathL*, *DcCathL* probe (Cy5; red) staining of the target transcripts in the gut cell nuclei; Merge, merged image. NC, negative control. 40x magnification.

FISH analysis indicated a concentration of the *DcCathL* in gut cell nuclei (**Figure 2**). The negative controls were incubated with Cy5-DcCathL probe, and no signal was detected. The quantification of the RF of the representative FISH gut analysis (**Supplementary Figure S3**) (N = 1 per condition) indicated an increase in the relative expression of DcCathL by approximately 3.3-fold higher in the infected insects compared to healthy insects.

### 3.2 Immunolocalization of the DcCathL protein

**Figure 3:**
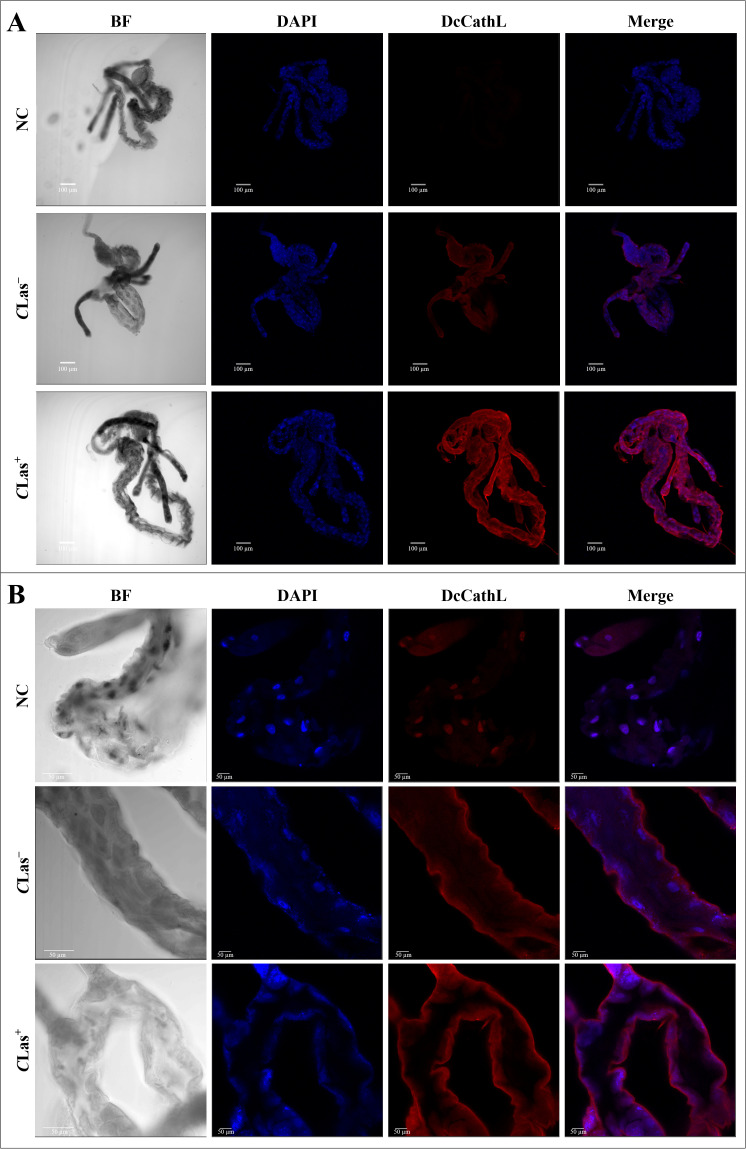
Immunostaining to detect DcCathL protein in gut tissues dissected from healthy (*C*Las^−^) and infected (*C*Las^+^) *D*. *citri*. **(A)** Visualization of the midgut under 10x magnification; **(B)** Visualization of the midgut under 40x magnification. BF, Bright field showing the morphology of the midgut tissue isolated from *D*. *citri;* DAPI, blue staining of the gut cell nuclei; DcCathL, DcCathL antibody + Alexa Fluor-568 (red) staining of the target protein in gut cells; Merge, merged image. NC, negative control.

**Figure 4:**
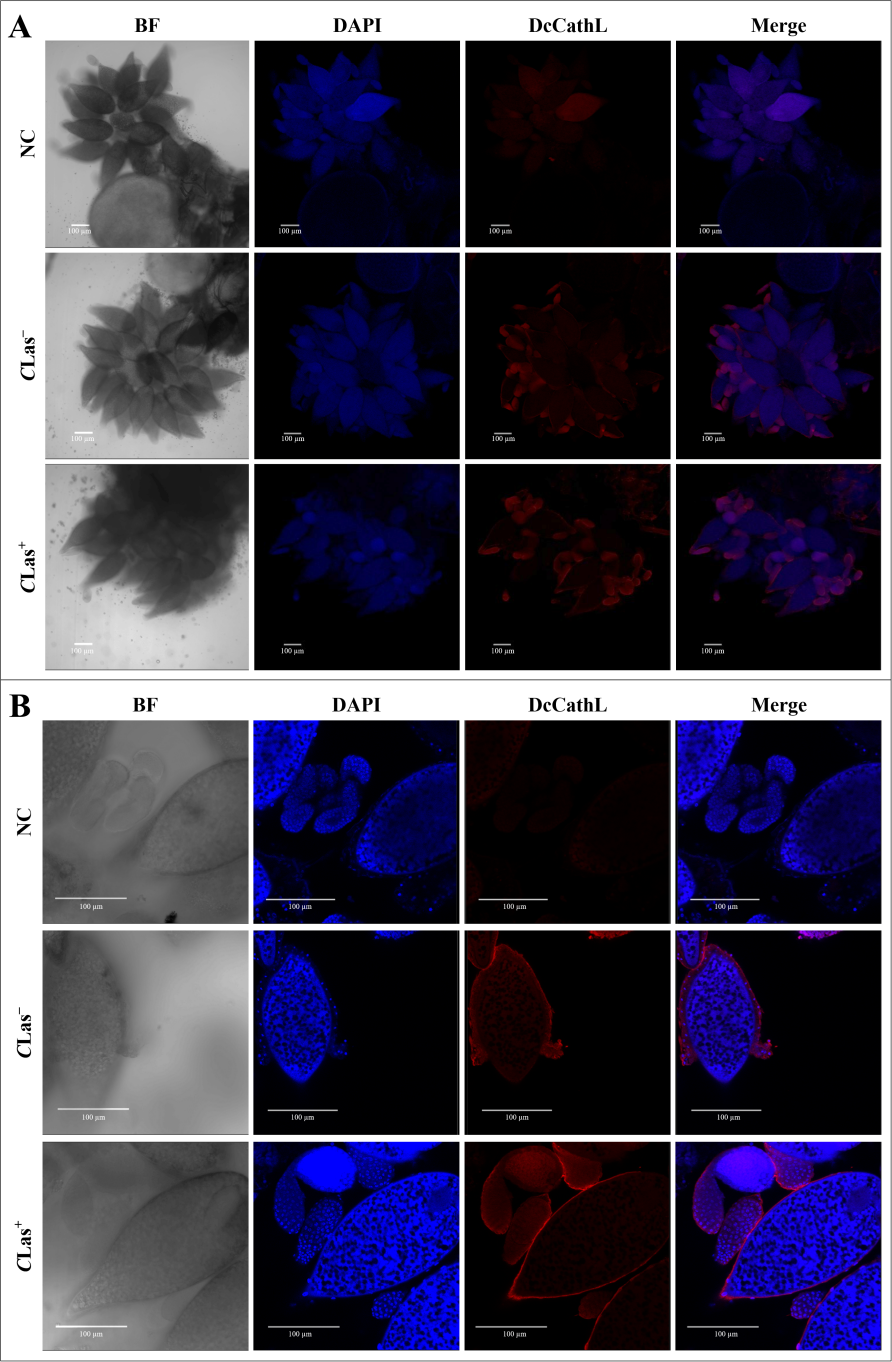
Immunostaining to detect DcCathL protein in ovary tissues dissected from healthy (*C*Las^−^) and infected (*C*Las^+^) *D*. *citri*. **(A)** Visualization of the ovaries under 10x magnification; **(B)** Visualization of the ovaries under 40x magnification. BF, Bright field showing the morphology of the ovary tissue isolated from *D*. *citri;* DAPI, blue staining of the ovary cell nuclei; DcCathL, DcCathL antibody + Alexa Fluor-568 (red) staining of the target protein in ovary cells; Merge, merged image. NC, negative control.

**Figure 5:**
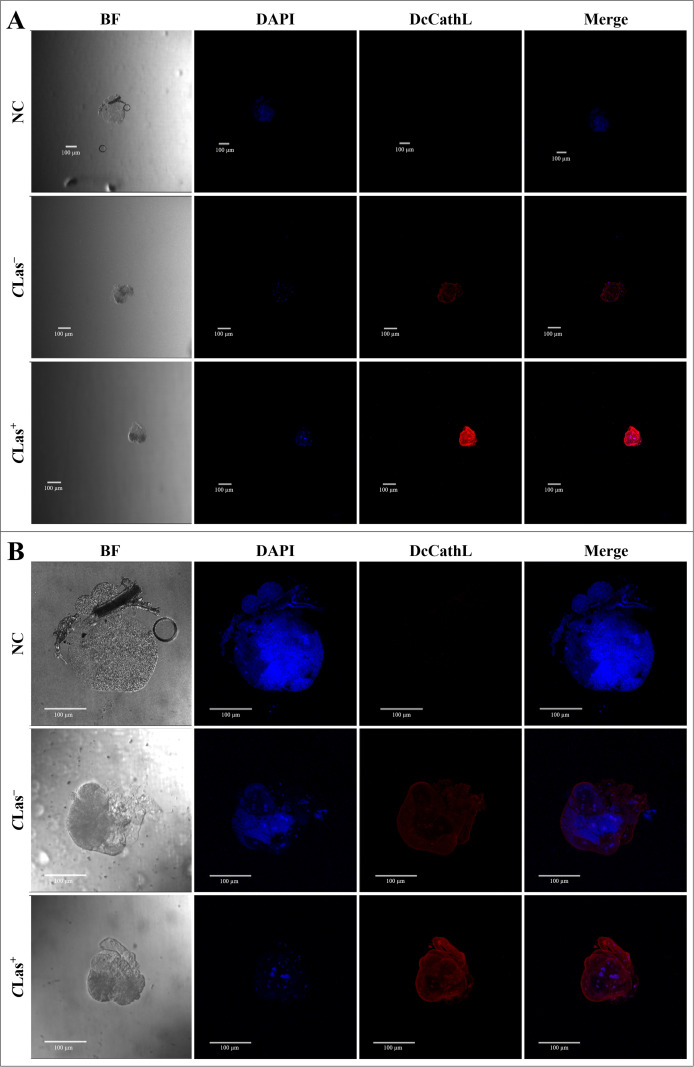
Immunostaining to detect DcCathL protein in salivary gland tissues dissected from healthy (*C*Las^−^) and infected (*C*Las^+^) *D*. *citri*. **(A)** Visualization of the salivary glands under 10x magnification; **(B)**: Visualization of the salivary glands under 40x magnification. BF, Bright field showing the morphology of the salivary gland tissue isolated from *D*. *citri;* DAPI, blue staining of the salivary gland cell nuclei; DcCathL, DcCathL antibody + Alexa Fluor-568 (red) staining of the target protein in salivary gland cells; Merge, merged image. NC, negative control.

Immunolocalization was used to visualize the localization of DcCathL protein in the midgut, ovary, and salivary glands of *D. citri* insects *C*Las^+^ or *C*Las^–^ (**Figures 3**–**5**). The representative quantification of the RF (N = 1 per condition) indicated a DcCathL-associated protein signal 4.81-fold higher in infected *D. citri* midguts than in uninfected midguts (**Figure 3A**; **Supplementary Figure S4A**). For the insect salivary glands and ovary, the relative expression was 1.38 and 1.1-fold greater in *C*Las^+^ than *C*Las^–^, respectively (**Figures 5A**; **Supplementary Figure S4C**; **Figures 4A**; **Supplementary Figure S4B**). The negative controls were incubated with an Alexa Fluor-568 secondary antibody, and no signal was detected. The DcCathL protein is located mainly in the membrane-peripheral zone of the cells (**Figures 3B**, **4B**, **5B**).

### 3.3 Artificial diet-feeding bioassays for *D. citri* nymphs and adults

**Figure 6:**
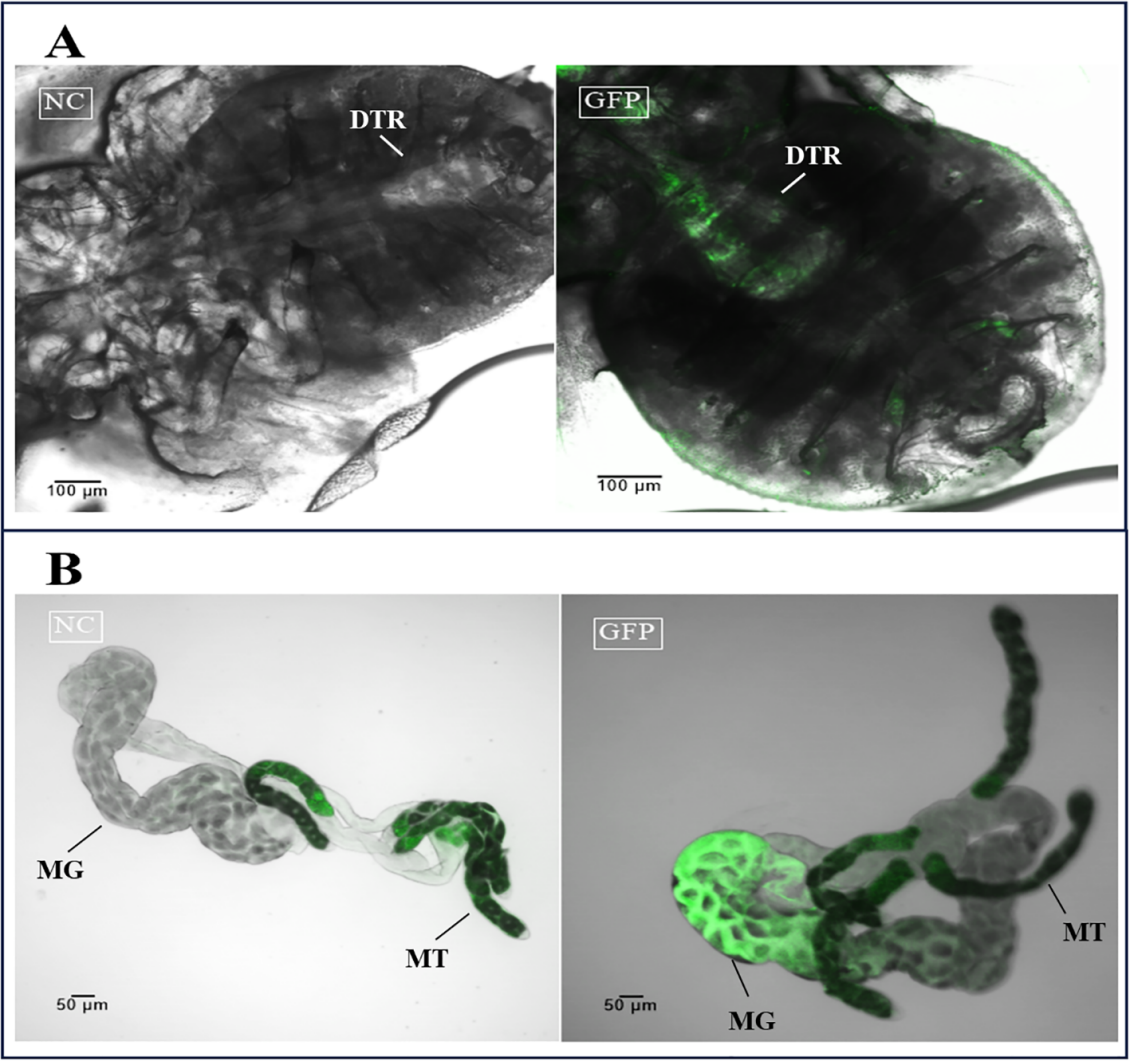
Uptake of recombinant GFP in the artificial diet of *D*. *citri.***(A)** rGFP detected in the nymph; 5^th^ instar; ventral view of the insect. **(B)** rGFP detected in the midgut dissected from *D*. *citri* adults. NC, negative control; GFP, green fluorescent protein; MT, Malpighian tubules (four appendages); MG, midgut; DTR, digestive tract region.

First, to confirm the efficiency of the artificial feeding assay for *D. citri* nymphs and adults, we tested the wipe-feeding (51) and parafilm M^®^ sachet-feeding assays (52), respectively, with a solution containing rGFP (or 0.1x PBS for the experimental control). The green color in the digestion tract of the dissected nymphs and adults’ guts indicates successful rGFP uptake during the feeding process in both the wipe-feeding and parafilm sachet-feeding tests (**Figures 6A, B**).

**Figure 7:**
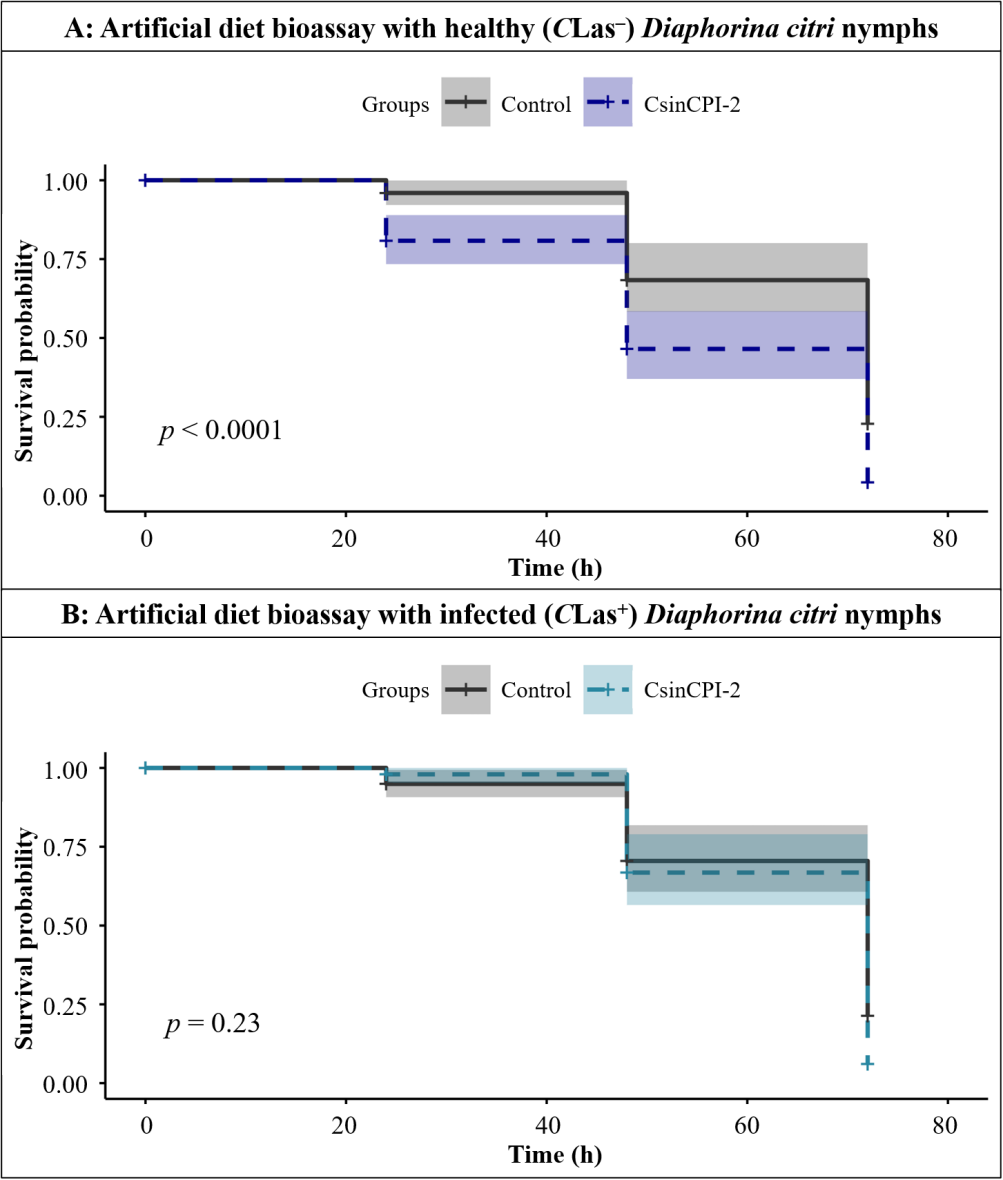
Cox proportional hazards regression showing the survival probability at 72 h of evaluation for *D*. *citri* nymphs in the wipe-feeding assay. **(A)** Survival probability of healthy nymphs in the presence or absence of the recombinant CsinCPI-2 protein after 72 h of the experiment; **(B)** Survival probability of infected nymphs in the presence or absence of the recombinant CsinCPI-2 protein after 72 h of the experiment. Statistical analysis was performed via RStudio software, with p<0.05.

**Figure 8:**
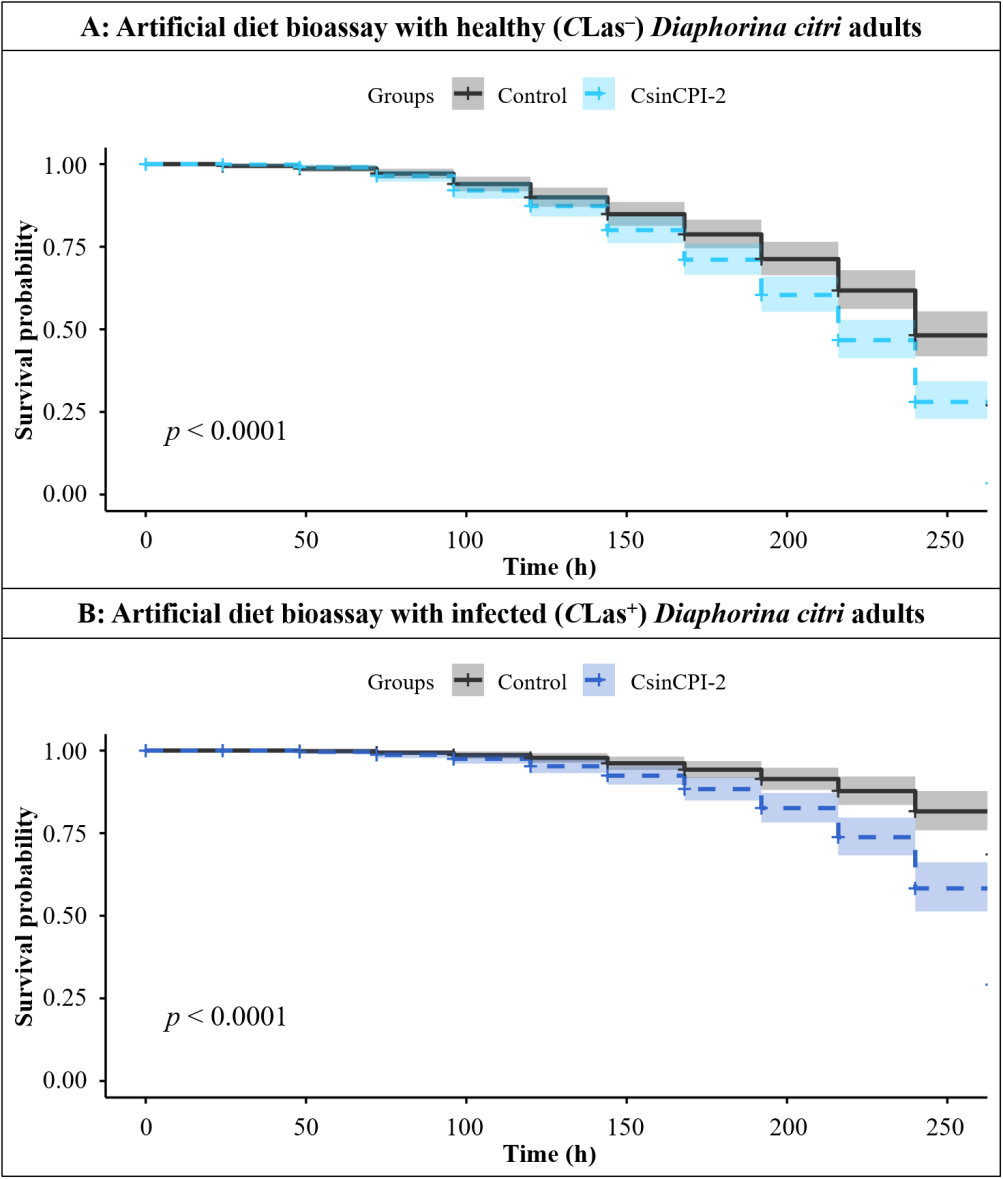
Cox proportional hazards regression showing the survival probability at 264 h of evaluation for healthy and infected *D*. *citri* adults. **(A)** Survival probability of healthy adults in the presence or absence of the recombinant CsinCPI-2 protein at 264 h of the experiment; **(B)** survival probability of infected adults in the presence or absence of the recombinant CsinCPI-2 protein at 264 h of the experiment; statistical analysis was performed via RStudio software with p<0.05.

Next, both artificial diet bioassays were used to evaluate the effects of the recombinant CsinCPI-2 protein on *C*Las^+^ and *C*Las^–^*D. citri* psyllids. For nymphs, a significant decrease in the survival probability for *C*Las^-^ nymphs fed with CsinCPI-2 protein was observed after 72 h (df = 1; *p* < 0.0001) (**Figure 7A**), whereas there was no statistically significant difference for the *C*Las-infected population (df = 1; *p* = 0.23) (**Figure 7B**). In adult assays, after 264 h, there was a significant increase in mortality in both healthy (df = 1; *p* = 0.0001) (**Figure 8A**) and infected (df = 1; *p* = 0.0001) (**Figure 8B**) populations fed with CsinCPI-2 cystatin.

## 4 Discussion

Cathepsin enzymes are associated with different developmental processes in insects, such as protein turnover, yolk protein mobilization, degradation, embryogenesis (23), ecdysis/metamorphosis (54), programmed cell death (55), digestion (24, 56) and immune defense (25). The knockdown of *DcCathB* and *DcCathL* in *D. citri* by CTV-RNAi led to a reduction in the number of developing oocytes and impaired ovarian maturation, which may be related to the downregulation of the *Vitellogenin* (*Vg*) gene (23). The significant impairment of *D. citri* development achieved by silencing the *Diaphorina citri* cathepsins (*DcCath*) genes (23) showed that these molecules are effective targets for limiting the spread of the *D. citri* vector. Thus, strategies to inhibit cathepsin activity constitute an interesting approach for managing HLB.

Previously, we reported that citrus cystatin proteins are potent inhibitors of *D. citri* cathepsin enzymes, with DcCathL being strongly inhibited by CsinCPI-2 cystatin (24, 47). Cystatins are defense proteins that act as peptidase inhibitors in various organisms, such as herbivorous insects (57). The activity of the inhibitors is due to their ability to form stable complexes with the target peptidases and to block access to the active site of the enzyme (32). The use of cystatins in the development of transgenic plants resistant to insects and pathogens is a widely used approach in which the corresponding genes encoding peptidase inhibitors are inserted into the plant genome, making it possible to obtain plants with improved traits (32, 58, 59). Furthermore, insects exposed to an artificial diet containing specific inhibitors for the main class of peptidases in their intestines may have retarded growth and development and a relatively high mortality rate (60). This finding reinforces that the interaction between DcCath enzymes and cystatins may affect HLB vector survival.

Given the importance of the DcCath enzymes in *D. citri* development, we focused our investigation on DcCathL as an essential target. First, the presence of *C*Las bacteria in *D. citri* positively affects *DcCathL* gene expression as previously shown (25), and we validated our assumption via RT–qPCR, which revealed increased *DcCathL* gene expression in *C*Las^+^*D. citri* adults (**Figure 1**), and FISH analyses, with an approximately three-fold increase in the relative fluorescence of *DcCathL* in the infected insect midgut (**Figure 2**; **Supplementary Figure S3**). In contrast to the *D. citri Cathepsin B-like* (*DcCathB*) reported to be 175-fold higher in the gut than in other tissues, indicating its digestive role (56), the expression of *DcCathL* in other tissues corroborates to its involvement in processes besides proteolysis (24). Thus, the upregulation of *DcCathL* gene expression in the gut in infected condition suggests that this enzyme may play a role in the immune defense system of *D. citri* (25), despite other functions. The midgut epithelium is the first physical barrier following oral intake and the abundance of digestive enzymes is responsible for the assimilation of nutrients from food (61). Therefore, the presence of DcCathL in the ovary and salivary glands also proposes significant involvement in embryonic development and the defense response, respectively (23, 25). Additionally, high expression of *DcCathL* was detected in the egg stage, reinforcing the role of this enzyme in *D. citri* embryonic development (24). Also, the knockdown of *DcCathL* in *D. citri* resulted in impaired ovarian maturation and led to a reduction in developing oocytes (23).

The immunolocalization of the DcCathL protein was investigated in the midgut, ovary, and salivary glands of *D. citri* (**Figures 3**–**5**). An approximately 1.4-fold increase in the relative fluorescence of DcCathL in the *C*Las-infected salivary glands compared to healthy tissue suggests basal expression of DcCathL in the gland (62) of *C*Las^–^ insects would mitigate the differences found in the relative expression of DcCathL in the gland of *C*Las^+^ insects (**Figure 5A**; **Supplementary Figure S4C**). However, the observed increase can possibly be attributed to the role of DcCathL in the transmission of the *C*Las, since the bacteria must reach the salivary glands to be successfully transmitted (25). A biofilm is formed in the midgut of *D. citri* and then *C*Las translocate to the hemolymph before reaching the salivary glands. Still, the exact mechanism by which the bacterium overlooks the insect’s immune system is unknown (14). Interestingly, high expression of a Cathepsin B-like protein, which is associated with triggering a host defense response, has been detected in the saliva and salivary glands of the aphid *Myzus persicae* (63). These findings reinforce the idea that DcCathL is involved in promoting the transmission of *C*Las by the psyllid (25).

Further, regarding the relative expression of DcCathL in the ovaries, no clear difference could be seen between *C*Las^+^ and *C*Las^-^ adults (**Figure 4A**; **Supplementary Figure S4B**), suggesting a slight impact of *C*Las infection in the tissue, with a concentration of the protein in the peripheral region, may being associated with the role in embryonic development (23–25). According to Xue et al. (37), the participation of Cathepsin L in eggshell formation has been observed in nematodes, indicating its participation in egg hatching (37).

Furthermore, in agreement with the results of the gene expression analysis, immunolocalization suggests a relative fluorescence of approximately five-fold higher of DcCathL in the *C*Las-infected midgut than in the *C*Las-free midgut (**Figure 3A**; **Supplementary Figure S4A**). This may be attributed to the diffuse distribution of the protein when evaluating the organ as a whole, with the digestive function (25). These findings indicate a greater impact of *C*Las infection on the expression of DcCathL in the gut compared to other tissues. Although, an increase in DcCathL expression under bacterial infection may reinforce its involvement in the immune system response of *D. citri* (23, 25).

In addition, immunolocalization revealed the DcCathL enzyme in the peripheral zone of the evaluated tissues (**Figures 3B**, **4B**, **5B**). Although cathepsins are associated mainly with lysosomal activity, the discovery that cathepsin is secreted in humans and remains active outside the lysosome has caused paradigm shifts (64). DcCathL possesses a signal peptide, indicating that it is a secreted protein in *D. citri* (25). Moreover, cathepsin was shown to be upregulated extracellularly under pathological conditions (64).

To test DcCathL inhibition *in vivo*, a recombinant citrus cystatin was used in an artificial feeding assay to confirm the deleterious effects of inhibiting the cathepsin enzyme present in *D. citri.* We observed that CsinCPI-2 cystatin significantly impaired the survival of healthy nymphs (*p* < 0.0001) (**Figure 7A**) but not infected (*p* = 0.23) (**Figure 7B**), and both healthy (*p* < 0.0001) and infected (*p* < 0.0001) (**Figures 8A, B**) adults.

Notably, *DcCathL* is expressed at higher levels in *D. citri* nymphs than in adults (25). This upregulation of *DcCathL* in the *D. citri* nymphal stage may be associated with *C*Las infection (25), considering that *D. citri* nymphs present the highest rates of *C*Las bacterium acquisition (8). At this stage, psyllids spend most of the time feeding (39), possibly, facilitating faster cystatin absorption than in adults, maximizing the harmful effects of CsinCPI-2 (**Figure 7A**), resulting the great mortality rate at this stage. Additionally, the nymph stage is generally more susceptible than the adult stage (51, 65, 66). As a result, the upregulation of *DcCathL* observed in nymphs (25), together with increased cathepsin expression when infected by *C*Las, likely overcomes competitive inhibition mediated by CsinCPI-2. In the absence of the bacteria, lower DcCathL expression has been demonstrated to maximize the effects of CsinCPI-2 inhibition, leading to increased mortality in this group (**Figure 7A**). In contrast, adults exhibit lower *DcCathL* expression levels compared to nymphs (25), suggesting a delayed cystatin-mediated inhibition, but effective against both *C*Las-infected and uninfected *D. citri* adults (**Figure 8**). This observation sheds light on the significant mortality rates that occurred in adult insects fed the same protein concentration only after an extended feeding period. These findings indicate that DcCathL plays an important role in *D. citri* and that citrus cystatins may be an effective tool to combat the HLB vector, especially in the nymphal stage, contributing to the reduction in *C*Las acquisition.

Our findings showed that the *C*Las bacterium can affect the relative gene expression of *DcCathL* as well as DcCathL protein expression, supporting the idea that these molecules are essential in the psyllid defense response to *C*Las (25). In addition to the presumed activity of the enzyme in the immune response of *D. citri*, which must be associated with its presence in the salivary glands, DcCathL has also been identified in the ovary, presumably with embryonic activity, and mainly in the midgut, indicating digestive activity. *C*Las-free nymphs were susceptible, although those infected with *C*Las were resistant to the effects of DcCathL inhibition. Additionally, both infected and *C*Las-free adults demonstrated sensitivity to the deleterious effects of the CsinCPI-2 protein. These interactions between *D. citri* and *C*Las are intricate, classifying them as complex HLB pathosystem. Furthermore, these findings are promising in the fight against HLB, where it is possible to infer that the interaction between DcCathL and CsinCPI-2 is capable of slowing the spread of the *D. citri* insect vector. Although further studies are still needed, our results suggest potential avenues for developing biotechnological solutions, such as a CsinCPI-2 protein spray solution or even transgenic plants overexpressing this protein, to slow down the spread of HLB.

## 5 Conclusion

In light of DcCathL expression coupled with the increased mortality in CsinCPI-2 cystatin-treated healthy nymphs and the mortality observed in both infected and uninfected adult psyllids, *DcCaths* can be considered important targets for the development of strategies for controlling the most serious citrus disease, particularly considering that adults are responsible for the dissemination of *C*Las bacterium to new citrus plants. Therefore, the increased mortality of nymphs can be an important alternative to control *D. citri*, since management at this stage should limit the acquisition of *C*Las. Hence, our findings demonstrated that the use of CsinCPI-2 could be a potential tool for the management of the *D. citri* insect by inhibiting DcCathL, opening up prospects for the development of an effective biotechnological solution to control the spread of HLB.

## Data Availability

The raw data supporting the conclusions of this article will be made available by the authors, without undue reservation.
