# Supplementary material for: Recombinant cystatin ingestion by Diaphorina citri reduces insect survival: insights into the HLB host–bacteria interaction (D. citri–CLas) focused on DcCathL studies
Source: Front Insect Sci. 2026 Feb 11;6:1700002. doi: 10.3389/finsc.2026.1700002 (PMC12933943; doi:10.3389/finsc.2026.1700002)
Supplement: Supplementary file 1 [file DataSheet1.pdf]

Supplementary Material

Figure legends

Table S1: *DcCathL* and *DcGAPDH* primer sequences

Primer	Sequence	Amplicon size (bp)	Accession number
**DcCathL-F**	5' CCACCATTGGACCCGTATCT 3'	85 bp	MN166228.1
**DcCathL-R**	5' TCAGGCTCGTAGTACACACC 3'		
**DcGAPDH-F**	5' GACACTCACTCCTCCATCTTT3'	96 bp	XM_008481619.1
**DcGAPDH-R**	5' GTATCCGTACTCGTTGTCATACC 3'		

Sequence of primers used in the gene expression analyses. The *DcCathL* primers were used for the target gene, and the *GAPDH* primers were used for the reference gene.

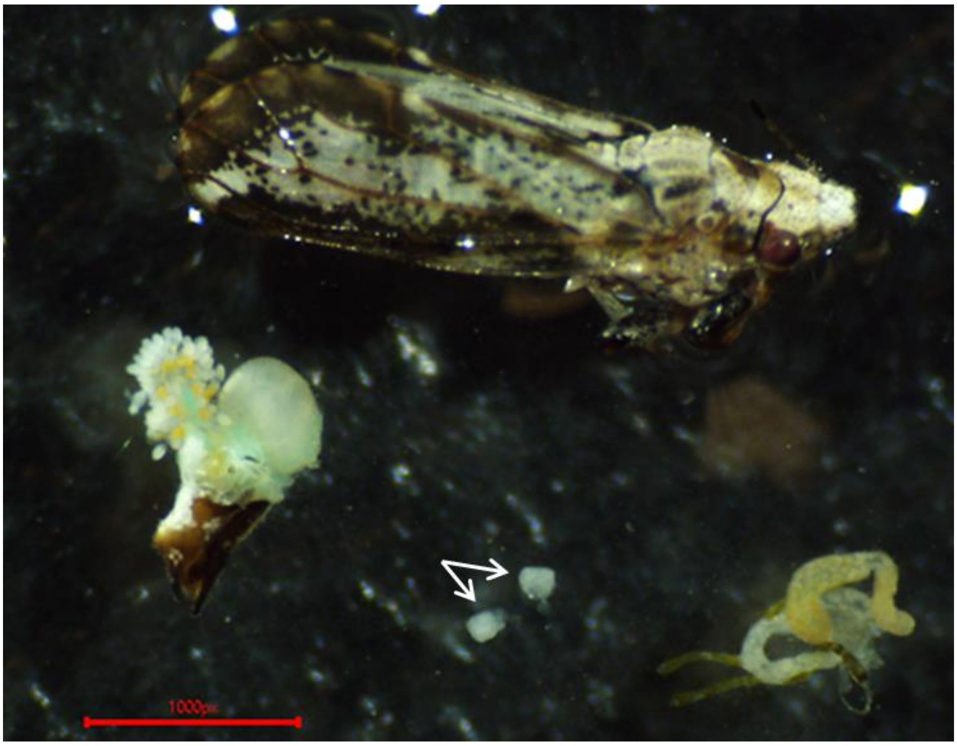

Figure S1- *D. citri* adult and organ dissection viewed under a stereomicroscope. The upper part is the adult insect; the bottom part is the ovary (on the left side), the salivary gland pair (white arrows); and the midgut (right side).

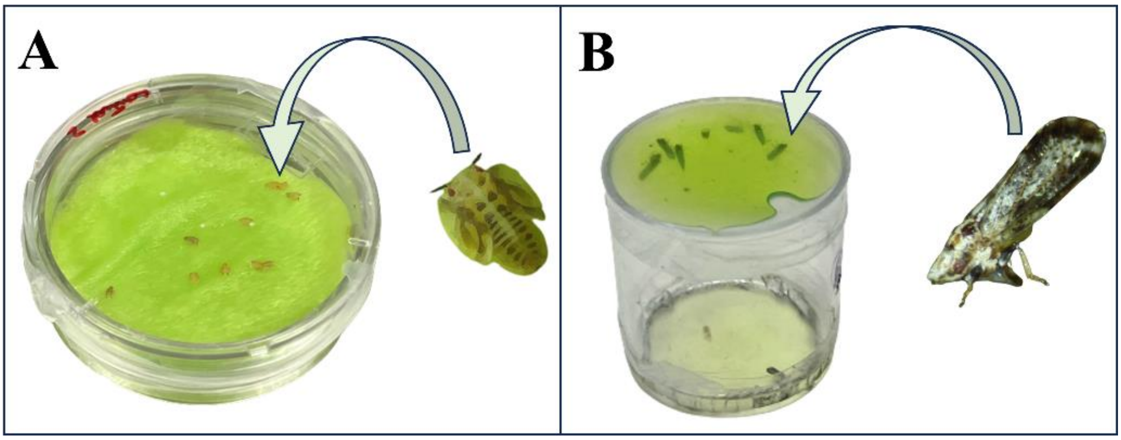

Figure S2- Schematic representation of the artificial diet bioassay for *D. citri*. **A:** Wipe feeding bioassay for 4th- and 5th-instar *D. citri* nymphs. **B:** Bioassay of *D. citri* adults fed Parafilm M® sachets.

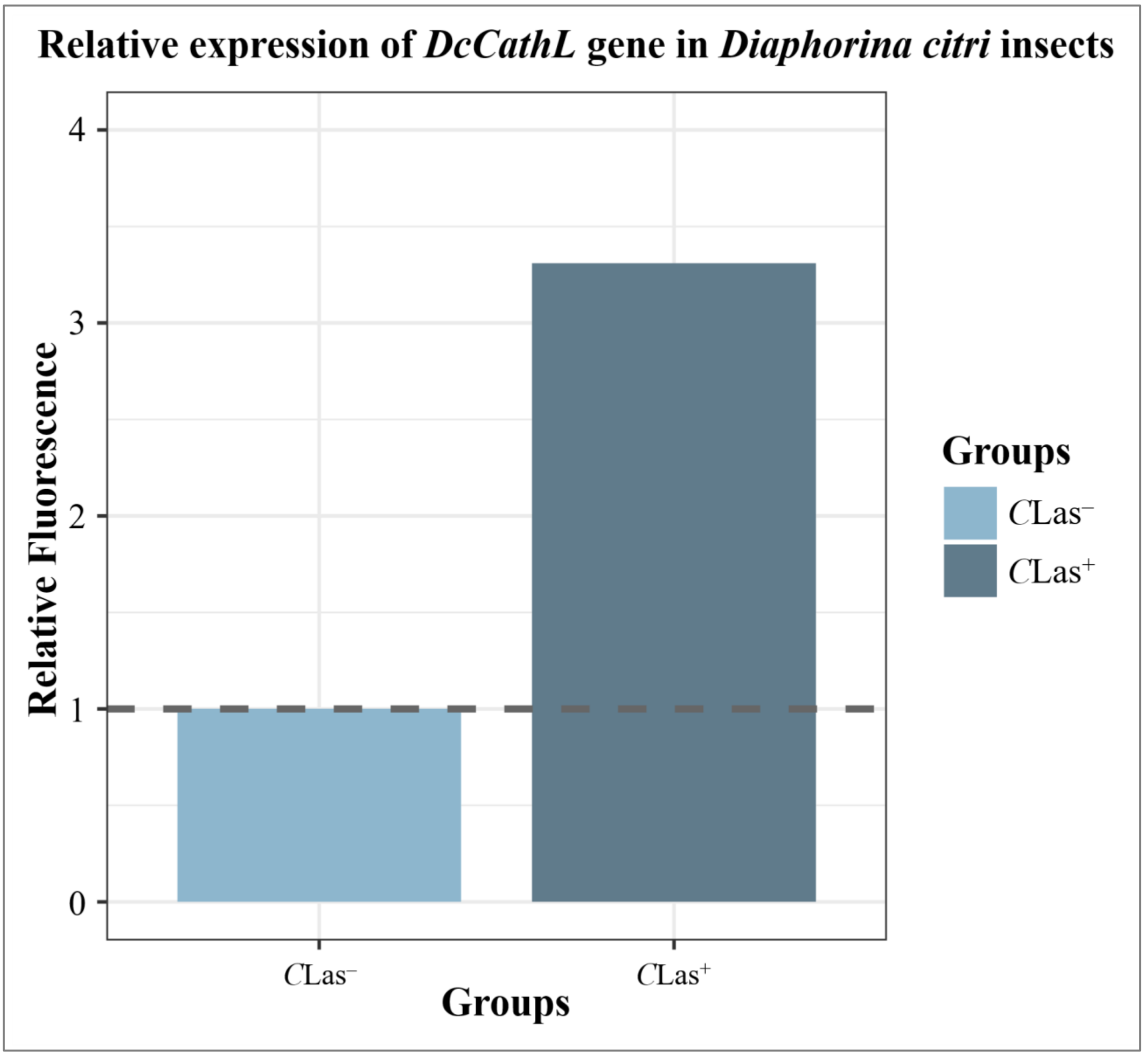

Figure S3: Relative expression of *DcCathL* gene in the *Diaphorina citri* insects. The fluorescence measurement of the *DcCathL* in the *D. citri* midgut was obtained after Fluorescence in situ hybridization (FISH) using the Cy5-DcCathL probe. The Relative

Fluorescence (RF) intensity was calculated based on the Corrected Total Area Fluorescence (CTAF) obtained via ImageJ (FIJI) (Schindelin et al. 2012; Ansari et al., 2013), and the raw data were analyzed via RStudio 4.4.1 (2024--06--14 ucrt).

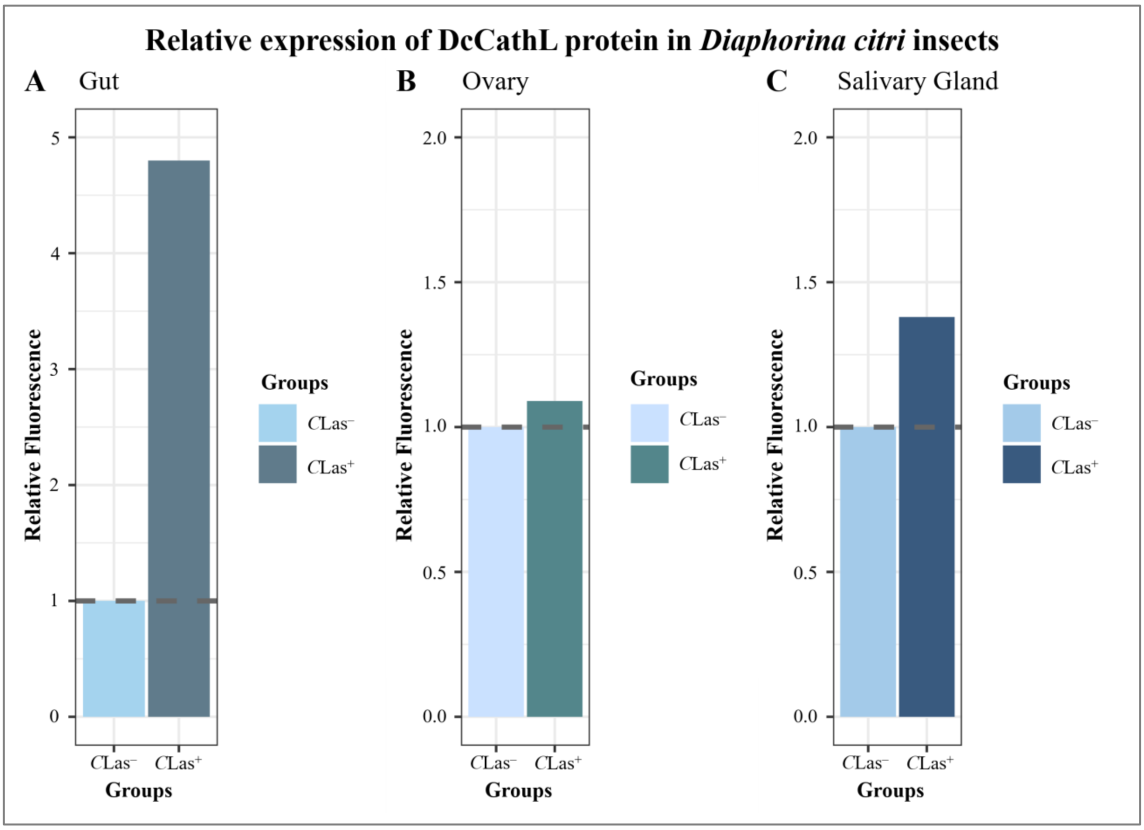

Figure S4: Relative expression of DcCathL protein in the *Diaphorina citri* insects. The fluorescence of the *D. citri* midgut (A), ovary (B), and salivary glands (C) were measured after immunolocalization of DcCathL with the anti-cathL antibody as the primary antibody and the goat anti-rabbit secondary antibody Alexa-Fluor 568 (Invitrogen, Waltham, Massachusetts, USA). The DcCathL Relative Fluorescence (RF) was calculated based on the Corrected Total Area Fluorescence (CTAF) obtained via ImageJ (FIJI) (Schindelin et al. 2012; Ansari et al., 2013), and the raw data were analyzed via RStudio 4.4.1 (2024--06--14 ucrt).
